# Editorial: Haemorrhoidal Disease: Old solutions and future perspectives Volume II

**DOI:** 10.3389/fsurg.2025.1714054

**Published:** 2025-11-17

**Authors:** Gianpiero Gravante, Veronica De Simone, Mario Trompetto, Arcangelo Picciariello, Renato Pietroletti, Petr Tsarkov, Gaetano Gallo

**Affiliations:** 1 Department of General Surgery, Azienda Sanitaria Locale ASL Lecce, Casarano, Italy; 2 Colorectal Surgery Unit, IRCCS San Raffaele Scientific Institute, Faculty of Medicine and Surgery, Vita-Salute University, Milan, Italy; 3 Department of Colorectal Surgery, S. Rita Clinic, Vercelli, Italy; 4 Department of Experimental Medicine, University of Salento, Lecce, Italy; 5 Surgical Unit, IRCCS de Bellis, Bari, Italy; 6 Department of Biotechnological and Applied Clinical Sciences, Proctology Unit, University of L'Aquila, L'Aquila, Italy; 7 Clinic of Coloproctology and Minimally Invasive Surgery, Sechenov University, Moscow, Russia

**Keywords:** hemorrhoidal disease, transperineal ultrasound (TPUS), patient-reported outcome measures (PROMs), post-operative pain, conservative treatment

## Introduction

Hemorrhoidal disease (HD) is as old as medicine itself. Descriptions of anorectal bleeding and prolapse can be traced to Egyptian papyri, Hippocratic texts, and medieval surgical treatises. For centuries, the primary solution was excision: crude at first, refined in the 19th and 20th centuries into the Milligan–Morgan and Ferguson hemorrhoidectomies (1). Excision became the benchmark against which all new treatments were measured, however pain, delayed recovery, recurrence, and functional disturbances remained significant concerns. More importantly, it reflected a surgeon-centred view of disease, with outcomes measured in terms of technical success rather than patient experience.

Today, the paradigm is shifting. The future of HD management lies *beyond excision*—towards approaches that combine scientific innovation with patient-centred outcomes. This includes new diagnostic tools such as transperineal ultrasound (TPUS), the use of patient-reported outcome measures (PROMs), minimally invasive or pharmacological innovations. The articles in this *Research Topic* illustrate this transition vividly. In this Editorial, we revisit the historical landscape of HD treatment, analyze the shortcomings of traditional approaches, and highlight promising innovations that point towards a patient-centred future.

### Historical overview of treatments

#### Conservative and medical measures

The earliest remedies for HD were conservative: dietary adjustments to reduce constipation, topical ointments to alleviate symptoms, and herbal mixtures to soothe bleeding. These remain part of the first-line management: phlebotonics and flavonoids have gained popularity for their ability to reduce bleeding and inflammation (2).

#### Conservative procedures

The mid-20th century marked a turning point with the advent of minimally-invasive procedures suitable in an outpatient setting. Rubber band ligation (RBL), introduced by Blaisdell in the 1950s (3), revolutionized management for grade II–III hemorrhoids thanks to its simplicity and favorable cost-effectiveness profile. Sclerotherapy, first described in the late 19th century, underwent periods of decline and revival (4). Early agents caused significant complications, but modern sclerosants have demonstrated improved safety and efficacy (4, 5). Recent studies support sclerotherapy as a minimally invasive alternative, particularly for high-risk patients (5, 6). More recently, several procedures—such as hemorrhoidopexy and even hemorrhoidectomy—have also been successfully transitioned from the inpatient to the outpatient setting in selected cases (7).

#### Excisional surgery

For advanced disease, excision dominated the 20th century. The Milligan–Morgan open hemorrhoidectomy and the Ferguson closed technique remain the reference procedures for grade III–IV HD, at the price of significant postoperative pain, prolonged recovery, and the risk of complications. The late 1990s brought Stapled Hemorrhoidopexy (SH), designed to reduce postoperative pain by lifting the hemorrhoidal plexus. Initially celebrated, SH fell under criticism for higher recurrence rates (8) and rare but severe complications (9, 10). Doppler-Guided Hemorrhoidal Artery Ligation (DG-HAL), often combined with mucopexy (11), emerged as another alternative, offering reduced pain but with mixed long-term efficacy (12, 13). Recent decades introduced numerous energy-based devices: infrared coagulation, radiofrequency ablation, and laser hemorrhoidoplasty. These aim to minimize tissue damage, speed recovery, and reduce pain.

#### Lessons from history

This historical progression reflects a constant tension: the trade-off between radical cure and patient comfort. Excision is effective but painful; outpatient measures are tolerable but prone to recurrence. What remains clear is that HD cannot be managed by a single “perfect” procedure, instead, a tailored approach is needed, integrating patient preferences, disease severity, and quality-of-life considerations (14). In example, a patient-tailored approach to the surgical treatment of hemorrhoids leads to equal satisfaction following hemorrhoidectomy, stapled hemorrhoidopexy or a combination of both (15).

### Patient-centred innovations

#### Advanced diagnostic perspectives: transperineal ultrasound (TPUS)

A major limitation in HD has been the reliance on subjective clinical evaluation. Unlike other fields, HD still relies heavily on clinical inspection and digital examination as no widely accepted imaging modality exists. This absence hampers research and patient-tailored decision-making. TPUS is emerging as a non-invasive modality capable of visualizing hemorrhoidal cushions and vascular flow in real time (16). TPUS provides several advantages: objective assessment—measurement of hemorrhoidal size and vascularization allows standardized documentation (16); therapeutic guidance—imaging can identify feeding arteries (16), informing decisions on sclerotherapy, embolization, or surgical intervention; follow-up monitoring—early detection of residual or recurrent disease without invasive examination (17); research standardization—TPUS metrics (vascular vs. scattered patterns, peak systolic flows) can serve as endpoints in clinical trials (16, 17). The advantage of TPUS is the easiness to learn, widespread diffusion, low cost, and availability in the pre-, intra- and postoperative setting (18).

Recent studies have demonstrated TPUS's ability to detect preoperative subtle changes and predict therapeutic outcomes (16–18). Integrating TPUS into routine practice bridges the gap between clinical impression and objective disease characterization, aligning diagnosis with patient-centred care.

#### Patient-centred outcomes

Traditional research endpoints—bleeding, prolapse, operative time—fail to reflect what matters most to patients. PROMs provide a structured approach to capturing the patient experience, including pain intensity and duration, symptom burden (bleeding, pruritus, soiling), functional impairment (i.e., work disruption), quality of life, and psychological impact (19, 20). Interventions producing similar anatomical outcomes may differ significantly in PROM-based satisfaction; their inclusion in trials and practice should be encouraged—a paradigm shift from surgeon-centred to patient-centred evaluation. PROMs (i.e., Sodergren, HSS, HDSS/SHS-HD) allow clinicians to quantify subjective outcomes, facilitating comparison between interventions from the patient's perspective and tailoring therapy to individual needs (21).

#### Innovative interventional approaches: rectal artery embolization

Recent advances in interventional radiology offer novel solutions. Rectal artery embolization (RAE) aims to reduce arterial inflow, inducing regression of the cushions without excision. Panneau et al. and Jiang et al. discuss efficacy and safety in small cohorts, demonstrating symptom relief and minimal complications (22, Jiang et al.). RAE may complement traditional surgery or replace it in selected patients.

#### Pharmacological horizons: targeted therapies for HD symptoms

Novel topical and systemic therapies are expanding the non-surgical landscape. Ai et al. targets HD-derived pruritus, Azhough et al. posthemorrhoidectomy pain using intradermal methylene blue, Yan et al. uncovered new potential applications for deoxycholic acid. These new symptom-control strategies add to the armamentarium to enhance patient comfort and improve satisfaction.

## Conclusions

HD carries the scars of centuries of excision-based practice. Today, however, we stand at the threshold of new horizons: imaging that reveals what was once unseen, outcomes that reflect the patient's voice, therapies that are less invasive and highly effective. Progress will not come from abandoning tradition, but from transcending it; the future of HD management is not merely about cutting less, but also caring more. Beyond excision lies the promise of safer, smarter, and truly patient-centred care.
